# A Rare Case of Systemic Lupus Erythematosus with Chylous Ascites and Chylothorax

**DOI:** 10.1155/2013/797696

**Published:** 2013-06-20

**Authors:** Dilek Ersil Soysal, Sezin Hizar Turan, Mustafa Ozmen, Mete Pekdiker, Mehmet Eren Kalender, Emrah Koc, Volkan Karakus

**Affiliations:** ^1^Department of Internal Medicine, Katip Celebi University, Ataturk Research and Training Hospital, 586/1 Sokak, Sezen Sitesi, Guzelbahce, 35340 Izmir, Turkey; ^2^Department of Rheumatology, Katip Celebi University, Ataturk Research and Training Hospital, 586/1 Sokak, Sezen Sitesi, Guzelbahce, 35340 Izmir, Turkey; ^3^Department of Hematology, Antalya Research and Training Hospital, 07100 Antalya, Turkey

## Abstract

During the course of the disease a patient with systemic lupus erythematosus (SLE) may develop inflammation of one or more serous membranes, resulting in pleural, peritoneal, or pericardial effusion. Chylous ascites and chylothorax have rarely been described in patients with SLE. Therefore, in parallel with the analysis of blood samples, detailed analysis of the effusions should be carried out. Supportive measures are often needed to relieve the symptoms of chylothorax or chylous ascites together with the treatment of the primary disease. The available literature had reported just 4 cases of chylous ascites and/or chylothorax in association with SLE, and this patient presented here is one of the rare cases apart from the reported ones.

## 1. Introduction

Chylous ascites or chylothorax is characterized by a milky-appearing fluid containing high levels of triglycerides [1, 2]. The causes of chylous ascites or chylothorax can be categorized as nontraumatic and traumatic [1–3]. Although the clinical features and causes of chylous effusions secondary to surgery or trauma are familiar to most clinicians, little is known about the incidence, etiology, and different clinical symptoms of atraumatic chylous effusions [1, 2]. The incidence of the combined occurrence of chylous ascites and chylothorax was from 9 to 55% of chylous effusions [2].

Systemic lupus erythematosus (SLE) is a chronic inflammatory disease that can affect the skin, joints, kidneys, lungs, nervous system, serous membranes, and other organs of the body [2]. Laboratory findings include antibody serologies that are positive for antinuclear antibodies (ANA) and frequently anti-Sm and anti-double stranded DNA (anti-dsDNA) [3, 4]. Besides ANA positivity, infrequently reported in the literature is finding of lupus erythematosus (LE) cells in body fluids [5, 6]. Although pleural and pericardial effusions are common in SLE and may occur at any time during the disease [5, 6], chylous ascites and chylothorax have rarely been described in patients with SLE [2, 3].

## 2. Case Report

We present a 61-year-old woman with a complaint of abdominal distension for a week. She has had a medical history of SLE and received 8 mg methyl prednisolon and 200 mg hydroxychloroquine daily for 10 years now.

The physical examination revealed decreased breathing sounds on the right and left lung bases and abdominal distension. 

Laboratory findings revealed white blood cells, 3.42 K/*μ*L with a lymphocyte count of 0.24 K/*μ*L (*N* = 0.8–4), erythrocyte sedimentation rate 6 mm/h, C-reactive protein 1.29 mg/dL (*N* = 0.0–0.8), serum albumin 2.8 g/dL (*N* = 3.5–5), LDL cholesterol 71 mg/dL (*N* < 130), HDL cholesterol 22 mg/dL (*N* > 35), and serum triglycerides 101 mg/dL (*N* = 40–150). Urine analysis was normal. Serology for hepatitis B, C, and HIV was negative. Antinuclear antibody was 1 : 1000 positive with a homogeneous pattern. Anti-dsDNA, anti-Sm, anti-histone, anti-RNP, anti-Ro, anti-La, anti-Scl-70, and antiphospholipid antibodies including lupus anticoagulant C and anticardiolipin were negative. Serum C3 was 66.2 mg/dL (*N* = 79–152), and C4 was 14.4 mg/dL (*N* = 16–38). Except for the elevated level of CA125 to 356 U/mL (*N* = 0–30.2 U/mL), tumor markers were negative. 

**Figure 1:**
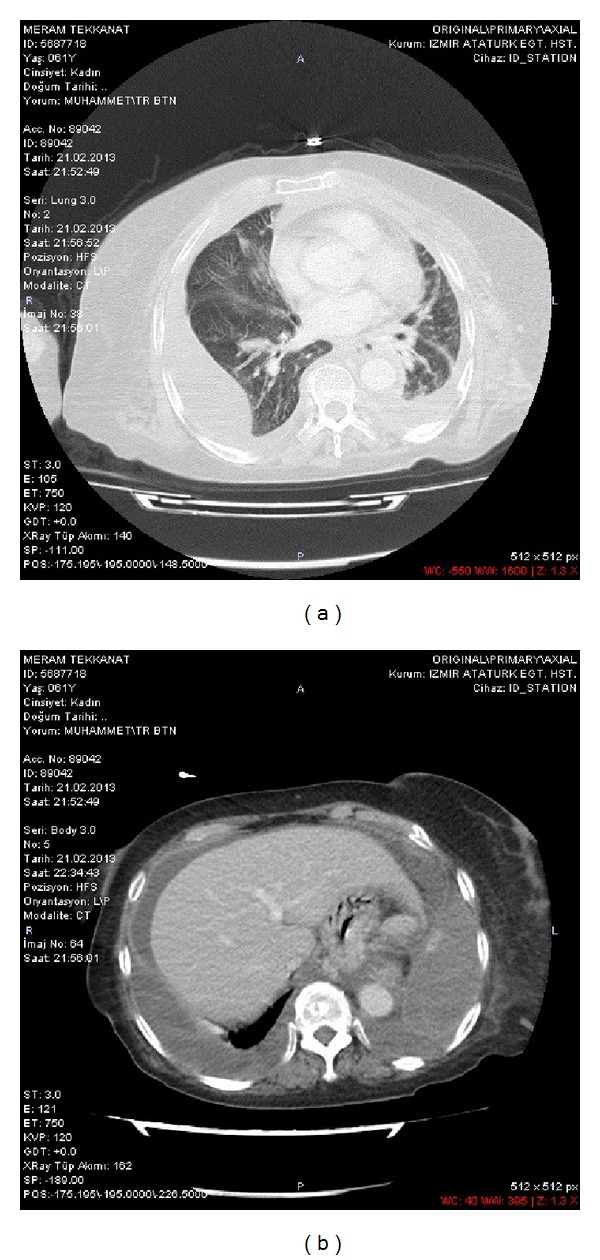
(a) A chest CT scan revealed bilateral pleural effusion with a partial compression at the left lung. (b) An abdomen CT scan revealed large amount of ascites of chylous type.

A chest CT scan revealed bilateral pleural effusions with a partial compression at the left lung base, an abdomen CT scan revealed large amount of ascites (Figures 1(a) and 1(b)), and an echocardiography revealed minimal pericardial effusion with preserved systolic function.

The ascitic fluid obtained by paracentesis had chylous appearance with the following biochemical values: leucocyte count 800/mm^3^ (96% lymphocytes), glucose 111 mg/dL, albumin 2.2 g/dL (serum-ascites albumin gradient 0.6), triglycerides 542 mg/dL, and lactate dehydrogenase 95 U/L (serum LDH 140 U/L). Antinuclear antibody was positive at a titer of 1 : 1000 with homogenous pattern and anti-dsDNA antibody was negative. The pleural fluid obtained by thoracocentesis revealed triglycerides of 315 mg/dL, ANA titer of 1 : 1000 with homogenous pattern, and negative anti-dsDNA antibody. Adenosine deaminase was 52.1 U/L. Polymerase chain reaction to *Mycobacterium tuberculosis* was negative. Gram stains of positive bacteria and mycobacterial smears were negative. Cultures of the peritoneal and pleural fluids for gram positive and negative microorganisms, Mycobacterium tuberculosis, and fungi were negative. Cytologic examination was consistent with nonspecific chronic inflammation and negative for the LE and malignant cells. 

The patient was treated with intravenous methyl-prednisolone of 1 mg/kg/day [2] for 15 days, and the dose was gradually tapered to 16 mg/day in 2 months. After discharge, the fluids regressed and the symptoms subsided in ten weeks. In addition to the corticosteroid therapy, she is using antimalarial drug hydroxychloroquine 400 mg/day now and is followed by a rheumatologist.

## 3. Discussion 

Several investigators have reported that, in comparison with younger patients, elderly patients with SLE have a more insidious onset of the disease and less common occurrence of the classic manifestations [7]. In our patient, the diagnosis of SLE depended on the revised 1997 American Collage of Rheumatology criteria [8] almost 10 years ago. On admission, she had combined occurrence of chylous ascites and chylothorax. She had neither clinical and laboratory findings of infection as tuberculosis, or protein-losing enteropathy, regional ileitis, cirrhosis, sarcoidosis, amyloidosis, thrombosis of the superior vena cava, heart failure, nephrotic syndrome, Behcet's disease, and malignancy nor a history of surgery or traumatic etiology for chylous ascites and chylothorax [2, 3]. 

Even though chyle has been defined as a noninflammatory fluid, inflammation is part of the mechanism responsible for the presence of this type of fluid in pleural and peritoneal cavities as in other forms of serositis associated in SLE [3]. A triglyceride concentration >110 mg/dL supports the diagnosis; a level <50 mg/dL excludes a chylous effusion [2]. It is possible that inflammation of the lymphatic vessels and cisterns provokes an increase in their endoluminal pressure and permeability of the walls leading to extravasation of chyle [3]. 

Age influences the serological manifestations of SLE [7]. Except for high titers of ANA, anti-dsDNA and LE cells were lacking in the patient's sera and ascitic and pleural fluids as well. In general, the serum ANA reflects disease activity and would be expected to be high in lupus pleuritis. A pleural fluid ANA titer of 1 : 160 or greater or a pleural fluid to serum ANA ratio of one or greater suggests that the pleurisy is secondary to active lupus [6]. Lower anti-dsDNA titres were found in patients with older onset SLE in one study, and similar results were reported in the other [7]. The presence of LE cells was correlated with either ANA or anti-DNA serologies [4]. Even though the finding of LE cells by cytopathology remains an important finding consistent with the diagnosis of SLE [5, 6, 9], their absence in pleural and ascitic fluids is not sufficient to discard lupus as the origin [3, 4]. 

In conclusion, during the course of SLE, a patient may develop inflammation of one or more serous membranes, resulting in pleural, peritoneal, or pericardial effusion. Chylous ascites and chylothorax have rarely been described in patients with SLE. Therefore, besides analysis of the blood samples, detailed analysis of effusions of the serous membranes should be carried out. 

We present a case of chylous ascites and chylothorax in association with SLE, and this patient presented here is one of the rare cases of the reported ones.
